# Is Hormone Replacement Therapy a Risk Factor or a Therapeutic Option for Alzheimer’s Disease?

**DOI:** 10.3390/ijms24043205

**Published:** 2023-02-06

**Authors:** Zoe B. Mills, Richard L. M. Faull, Andrea Kwakowsky

**Affiliations:** 1 Centre for Brain Research, Department of Anatomy and Medical Imaging, Faculty of Medical and Health Science, University of Auckland, Auckland 1023, New Zealand; 2 Pharmacology and Therapeutics, School of Medicine, Galway Neuroscience Centre, Ollscoil na Gaillimhe—University of Galway, H91 W5P7 Galway, Ireland

**Keywords:** Alzheimer’s disease, memory, dementia, cognition, estrogen, estradiol, hormone replacement therapy

## Abstract

Alzheimer’s disease (AD) is a progressive neurodegenerative disorder that accounts for more than half of all dementia cases in the elderly. Interestingly, the clinical manifestations of AD disproportionately affect women, comprising two thirds of all AD cases. Although the underlying mechanisms for these sex differences are not fully elucidated, evidence suggests a link between menopause and a higher risk of developing AD, highlighting the critical role of decreased estrogen levels in AD pathogenesis. The focus of this review is to evaluate clinical and observational studies in women, which have investigated the impact of estrogens on cognition or attempted to answer the prevailing question regarding the use of hormone replacement therapy (HRT) as a preventive or therapeutic option for AD. The articles were retrieved through a systematic review of the databases: OVID, SCOPUS, and PubMed (keywords “memory”, “dementia,” “cognition,” “Alzheimer’s disease”, “estrogen”, “estradiol”, “hormone therapy” and “hormone replacement therapy” and by searching reference sections from identified studies and review articles). This review presents the relevant literature available on the topic and discusses the mechanisms, effects, and hypotheses that contribute to the conflicting findings of HRT in the prevention and treatment of age-related cognitive deficits and AD. The literature suggests that estrogens have a clear role in modulating dementia risk, with reliable evidence showing that HRT can have both a beneficial and a deleterious effect. Importantly, recommendation for the use of HRT should consider the age of initiation and baseline characteristics, such as genotype and cardiovascular health, as well as the dosage, formulation, and duration of treatment until the risk factors that modulate the effects of HRT can be more thoroughly investigated or progress in the development of alternative treatments can be made.

## 1. Introduction

Ageing is an inevitable biological process that contributes to the accruement of cellular damage and dysfunction. As the global population and average life expectancy continue to increase, the impact of age-related diseases on public health systems is becoming exponentially overwhelming. As a result, the demand for treatment is growing, yet therapeutic options remain limited [1]. Idiopathic AD is the leading form of dementia in the Western world, presenting as mild cognitive impairment (MCI) in the prodromal stages of the disease and progressing to severe and debilitating symptomatology. In these late stages, the severity of neuronal degeneration limits the efficacy of current treatment options and requires patients to seek advanced palliative care. With the risk of developing AD increasing with age, doubling every five years after 65, estimates of global dementia cases predict a near tripled demographic size by 2050 [2]. Women account for two-thirds of this estimate, reflecting the 2–3 times increased risk of developing AD than men, where symptoms of cognitive decline often present as more severe in women and progress into the late stages of the disease more rapidly [3,4]. Prioritizing the identification of factors responsible for the overrepresentation of women and determining approaches to decrease this elevated risk could have significant implications for the imminent increase in global AD incidence.

Historically, there has been substantial gender bias in research involving the predominating and exclusive use of male animal models [5]. This prejudice stems from the justification that sex-specific differences in anatomy and physiology, such as the menstrual cycle, introduce confounding variables. This dangerous precedent has led to a reduction in female-focused biomedical findings, which has restricted progress toward understanding how sex-specific differences impact disease etiology like in AD [6]. One contrasting difference between men and women is apparent in endocrinologic function and fluctuations in adult hormone levels [7]. Estrogens are the main steroidal female sex hormone, predominantly synthesized upon stimulation of primary follicle development, functioning to control ovulation as one of several physiological roles. Unlike male sex hormones, where androgen expression remains relatively constant throughout adulthood, the natural exhaustion of the primordial female follicle pool during menopause causes the cessation of estrogen production and the rapid decline in endogenous hormone levels. Both androgens and estrogens are neuroactive and are known to have significant neuroprotective effects; however, as a result of the loss of female neuroprotective factors, it has been hypothesized that this profound change in the estrogenic environment during menopause may contribute to an increased vulnerability in women to develop AD [8,9,10].

Hormone replacement therapy (HRT) prescription for peri- and postmenopausal women has historically been used to supplement endogenous estrogen levels and alleviate undesirable vasomotor and menopausal symptoms, including depression, irritability, hot flashes, perspiration, and loss of bladder control [11]. In 1994, Henderson et al. [12] revealed that women diagnosed with AD were significantly less likely to have used HRT to treat their menopausal symptoms than healthy women. Following on from this discovery, other early epidemiological studies, which focused on women’s health throughout the 1990s conferred, reported decreased incidence of AD among women who took HRT compared to those who did not [13,14,15,16,17,18,19,20]. Metanalysis of collated results from studies during this era showed that the risk of developing AD in HRT users decreased by almost 30%, providing strong evidence to substantiate the beneficial claims of HRT on cognition [21,22]. For the remainder of the decade, interest in HRT as a neuroprotective treatment became increasingly popular, provoking laboratory research to investigate the underlying mechanisms of estrogens and instigation of clinical trials to further address this hypothesis.

The Women’s Health Initiative Memory Study (WHIMS) [23] recruited a subset of patients from the Women’s Health Initiative Study (WHI) and is the largest-scale randomized control trial to date that assessed the effect of HRT on dementia and cognitive impairments. Eligible participants were dementia-free and over 65 years old. A total of 4532 participants were randomized and received rather continuous combined HRT, or placebo [24], while an additional 2974 hysterectomized women received continuous unopposed HRT, or placebo [23]. After 4 years, the collected data showed that HRT exposure caused a twofold increase in the risk of dementia diagnosis, exhibiting exacerbated cognitive decline compared to controls. At the time, the results of WHIMS were particularly controversial, as they directly conflicted with evidence demonstrating the benefits of estrogen on cognitive health. Today, these results have left a profound uncertainty surrounding the clinical recommendation for postmenopausal women, which has persisted in the literature and as public opinion nearly two decades later.

This review focuses on evaluating critical differences in clinical and observational studies that have investigated the impact of estrogens on cognition or attempted to answer the prevailing questions regarding the use of HRT as a therapeutic option for AD (Table 1). The articles were retrieved through a systematic review of the databases: OVID, SCOPUS, and PubMed (keywords “memory”, “dementia,” “cognition,” “Alzheimer’s disease”, “estrogen”, “estradiol”, “hormone therapy” and “hormone replacement therapy” and by searching reference sections from identified studies and review articles). The papers returned from the search were then manually screened for relevance. This review aims to present the relevant literature available on the topic and discuss the mechanisms, effects, and hypotheses that contribute to the conflicting findings of HRT in the prevention and treatment of age-related cognitive deficits and AD.

## 2. Estrogens and Neuroprotection

**Figure 1:**
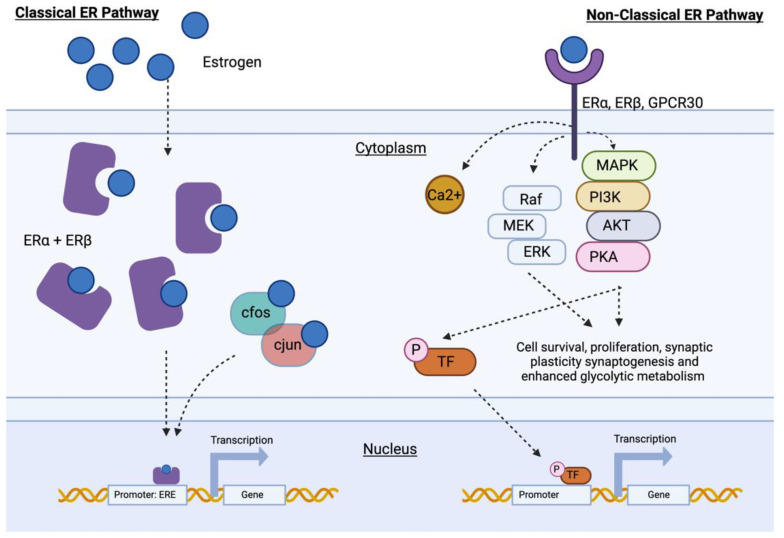
Schematic diagram showing the proposed mechanism of classical and non-classical estrogenic action. Activation of the classical ER pathway (**left**) involves nuclear ER found in the cytoplasm directly binding to EREs within target gene promotor regions. Transcription factors, such as c-fos and c-jun, facilitate binding of the nuclear ER to begin gene transcription. The non-classical pathway (**right**) is facilitated through membrane-bound ER. Upon ligand binding, intracellular protein kinase cascades result in changes to the intracellular environment and activation of independent transcription factors. Abbreviations: ERα: estrogen receptor α; ERβ: estrogen receptor β; ERE: estrogen response element; ERK: extracellular regulated kinase; GPCR30: G protein-coupled receptor 1; MAPK: mitogen-activated protein kinase; PI3K: phosphatidylinositol 3-kinase/Akt signaling; PKA: protein kinase A; Raf/MEK: mitogen-activated ERK kinase signaling; TF: transcription factor.

The biosynthesis of estrogens requires the conversion of cholesterol and subsequent aromatization of intermediate androgens [77]. Dictated by the availability of enzymes to facilitate these reactions, the main bioactive estrogen products produced by premenopausal, non-pregnant women are 17β-estradiol (Estradiol, E2) and Estrone (E1) [78]. As steroid hormones, estrogens are secreted into the circulation bound to hormone-binding globulins and distributed throughout the body. The classical understanding of estrogen action is that upon entering the cell via passive mechanisms, the stereospecific ligand binding of the estrogen family to nuclear ligand-gated receptors causes dimerization and subsequent transport of the receptor complex into the nuclear compartment to facilitate genomic responses (Figure 1). Although the primary estrogen receptor (ER) isoforms, ERα and ERβ, are predominantly expressed in reproductive tissues, the identification of both receptor species in the cortex, hypothalamus, and hippocampus indicates a local role for estrogens in the brain [79,80,81].

Altering genomic mechanisms is a slow yet robust method for influencing protein-synthesis-dependent cellular processes. The estrogen response element (ERE) is a specific sequence embedded in the promoter region of DNA, with a high binding affinity for ER complexes, allowing estrogen to act as a transcriptional modulator (Figure 1). Through transactivation and modulation of both transcriptional machinery and cofactor activity, estrogens can directly regulate the expression of genes with a high level of specificity [82]. The activator protein-1 (AP-1) site in immediate early genes, such as *c-fos* and *c-jun*, also facilitates estrogen’s ability to manipulate protein–protein interactions and feedback to higher-order loops of gene expression [83]. Using genomic mechanisms, estrogen can directly induce the production of structural proteins, neurotrophins, and anti-apoptotic factors that are required in synaptogenesis, proliferation, and synaptic plasticity [84,85]. Estrogenic activity can also down-regulate genomic mechanisms, suppressing the expression of apoptotic and programmed cell death genes, such as *Bcl-2,* to promote cell survival [86,87,88,89,90].

Recognition of an alternative mechanism of estrogenic action was acknowledged following the discovery and demonstration that rapid activation of intracellular responses could be achieved by transduction of signals through plasma-membrane-associated ER (Figure 1) [91]. These ERs include ERα and ERβ, which localize to the membrane of both nuclear and non-nuclear compartments of a variety of cell types, in addition to several other species of estrogen-binding receptors, including ER-X [92] and G protein-coupled estrogen receptor 1 (GPCR30) [93]. Stimulation of these membrane-bound receptors by extracellular estrogen causes local changes in ion activity and increased calcium mobilization. Coupled signals from the cell surface can activate cascades of secondary messengers, triggering several potent pathways associated with maintaining and developing healthy neural networks. Activation of the PI3K (phosphatidylinositol 3-kinase)/Akt (protein kinase B) pathway mediates cognitive health by promoting proliferation, angiogenesis, catalyzing pro-survival kinases, increasing metabolism, and efficient bioenergetic processes [94]. The Raf/MEK (mitogen-activated ERK kinase)/ERK (extracellular-related kinase) pathway has also been shown to allow indirect activation of genomic mechanisms and mediate the E2 neuroprotective response to glutamate toxicity in AD [95,96].

In animal models of AD, estrogenic signaling provides neuroprotective relief against the pathological hallmarks of the disease. For example, estrogen is well documented in its capacity to influence the fate of the amyloid precursor protein by upregulating the non-amyloidogenic pathway and increasing enzymatic activity to reduce the abnormal accumulation of the amyloid-beta (Aβ) peptide to form senile plaques, one of the major hallmarks of the disease [21,89,97,98,99,100,101,102]. Additionally, ERα has been observed to colocalize and bind to tau proteins to prevent hyperphosphorylation and tangle formation [103,104], provide resistance against glutamate toxicities and inflammation [105,106], while promoting synaptic plasticity and efficient glycolytic metabolism [107,108]. Ovarian hormone deprivation has also been linked to accelerated epigenetic and cellular senescence due to the loss of estrogen-mediated retention in telomere length and lower telomerase activity [109,110].

Additionally, estrogens have been shown to influence expression of AD genes. In female rhesus macaques the target genes of estrogen were identified using a database of over 17,000 diverse gene sets [111]. The findings of the study demonstrated that among the 1027 genes in the data set, which have been previously associated with AD, 84 of these genes were found to be regulated by estrogen, a conservative number estimated using a fold change value of >2. Included in this capture was the *APOE* gene, which is associated with genetic predisposition to AD (discussed in Section 4), with a further 10 overlapping genes functioning within the mitochondrial ‘bioenergetic’ pathway, supporting estrogen’s wider role in preventing vascular dysfunction, which is sometimes theorized to precede even disruptions to amyloid in the pathological cascade of AD. Other notable genes that contain ERE include brain-derived neurotrophic factor (BDNF) [112], explaining a mechanism by which estrogen upregulates BDNF protein availability, as well as the estrogenic inhibition of the *BACE1* gene encoding the amyloidogenic β-secretase enzyme, constraining Aβ synthesis [113]. Polymorphisms of genes encoding enzymes and proteins primarily responsible for the functionality, biosynthesis, and turnover of estrogen have also been identified to increase risk of AD in carriers (specifically: *GSTM3*, *GSTT1*, *ChAT*, *NEP*, *GSTM1*, *GSTO1*, *GSTO2*, *ERS2*, *BuChE* and *ERS1* with many others playing secondary modulatory roles) [114]. Hence, consideration of the classical mechanism of estrogenic action is paramount in deciphering the complex genomics of AD and HRTs clinical application.

Under laboratory conditions, estrogens have displayed overall beneficial effects at the molecular and cellular levels and are capable of promoting general health, in addition to protecting against the onset of specific pathologies. However, understanding the full therapeutic potential of estrogens against disease is complex and still under investigation due to the multitude of neuroprotective pathways that the classical and alternative mechanisms of estrogens can activate [115,116,117,118].

## 3. Critical Window Hypothesis

The dichotomy between the observations of early epidemiological studies and the findings of WHIMS is perplexing, considering the theoretical utility of estrogen, which has been substantiated by laboratory-demonstrated evidence. Reconsideration of the WHIMS study population has raised concerns about the generalizability of the results to realistic users of HRT. Due to the increased risk of AD in older women, WHIMS hypothesized that this age group would benefit the most from HRT treatment. However, as the average age of menopause onset occurs at 51, the estrogen levels of women in WHIMS would have been depleted for ~14+ years [119]. Sherwin and Maki, two experts in the field, were the first to offer the ‘critical window hypothesis’ as a possible explanation for these incongruent findings, proposing that HRT effectivity is critically dependent on a temporal window of administration that exists shortly after the menopausal transition [65,120,121,122,123,124].

Initial support for this hypothesis came from the re-examination of the WHIMS [121] and ‘Cache County Study’ [20,26] data. In contradiction to the original findings of these studies, which reported an increased risk of AD in women prescribed HRT, stratification of the data into current and former HRT users indeed revealed a lower risk of AD in favor of previous use. Furthermore, an ancillary Cache County study, re-assessed how age of initiation modulated AD risk. Using this method, statistical analysis revealed that estrogen supplementation did have a protective effect, but only in women who started treatment within five years of menopause onset [26]. Similarly, the ‘Longitudinal Study of Aging in Women’ (LAW; [125]) reported neither a beneficial or adverse effect on memory, while re-examination showed that those who started HRT within three years of menopause scored higher on cognitive tests than those who never used HRT, and significantly higher than users who initiated HRT later in life [28].

In order to directly address the critical window hypothesis, several studies implemented a design that allowed comparison of the effect of HRT between age groups. The ‘Multi-institute research in Alzheimer’s epidemiology’ study (MIRAGE; [126]) analyzed participants using three age groups: 50–63, 64–71, 72–99. Lower relative risk of AD in HRT users were found in all groups; however, the greatest benefit was experienced by the youngest tertile, an effect that was weakened as age increased. Studies using broader cognitive and neuropsychiatric outcome measures have also shown similar results. The ‘Kaiser Permanente Study’ [127] also demonstrated a lower incidence of AD in those who were HRT users during midlife, but increased in late life users. The ‘Study of Women’s Health Across the Nation’ (SWAN; [30]) demonstrated that the initiation of HRT before the final menstruation period was more beneficial for cognitive health than those who initiated after this time. Furthermore, participants in the study of ‘Research into Memory, Brain Function, and Estrogen Replacement’ (REMEMBER; [31]) who initiated HRT after the age of 56, performed significantly worse than both young users and never-users of HRT.

Like WHIMS, several other randomized control trials (RCTs) published in the early 2000s used specified age cut offs for their study population, allowing conclusions and comparisons to be made based on study demographics. Three RCTs with a mean age between 66 and 71 failed to show any relationship between HRT and changes in cognitive function [43,44,45], while an RCT with a younger study population (mean age 51) showed benefits in several different cognitive tasks after just 21 days of treatment [46]. The ‘WHIMS of Younger Women’ (WHIMSY; [47,48]) compared HRT in participants ages 50–55 to 65–79 who had no prior use of HRT. Older participants who used HRT exhibited long-term declines in global cognitive scores, working memory, and executive functioning. Furthermore, Girard et al. [49] and Dunkin et al. [50] demonstrated that if initiated close to menopause, HRT caused substantial improvements in cognitive control. Together, these studies provide strong evidence supporting the existence of a critical timeframe soon after menopause at which estrogenic treatment is beneficial. However, the exact parameters of this period and underlying mechanism remain unclear.

Animal studies have attempted to provide rationale for the critical window hypothesis using ovariectomized or older postmenopausal rodents to replicate the estrogen deprivation experienced after menopause. After long periods of estrogen depletion, the beneficial effects of estrogen in the restoration of synapse or spine density, hippocampal responsiveness, and synaptic plasticity mechanisms were absent [87,88,128,129,130,131,132,133]. In human studies, surgical removal of the ovaries at an early age is associated with a 70% increased risk of AD unless hormonal intervention is given [134]. The ‘Singapore Chinese Health Study’ [51] investigated the influence of female reproductive factors on cognition. They found that HRT users had 39% lower odds of cognitive impairment. Additionally, the age of menopausal transition also appears to be a determining factor for neurocognitive performance, with those who were more recently menopausal displaying enhanced capacity for improvement [52]. Collectively, these studies indicate that the premature or prolonged loss of estrogens results in the loss of HRT effectiveness as a treatment in addition to increasing the risk of AD.

The degree to which age modulates drug effects is still a relatively novel concept and likely to be a complex repercussion of several adaptations to cell functionality. Biological plausibility of these phenomena occurring in estrogen-mediated pathways has been determined by in vitro studies, where aged neural tissue becomes unresponsive to changes in the estrogenic environment. One possibility for this change is that the extended period of depleted estrogen levels cumulatively results in a loss of ER [135]. ERα can localize to the membrane surface through interaction with protein complexes and lipid rafts [136]. Zhang et al. [137] demonstrated that the rate at which ERα dissociates from these rafts to undergo CHIP-mediated ubiquitination and proteasomal degradation during long-term estrogen deprivation is increased. Decreased ER retention may be triggered as a consequence of decreased genomic suppression of the *BcL-2*-associated gene *Bag-1*, which functions to regulate the transport of ERα for degradation in this process. Preliminary evidence reported by Bean et al. [138] has also demonstrated that genomic upregulation of ERα expression is sufficient to restore estrogen’s beneficial effects on synaptic plasticity mechanisms in postmenopausal animals. This study indicates that receptor expression is one limiting factor of the temporal window and insinuates that manipulation of ERα expression could potentially influence a reopening or increase in the duration of this period.

## 4. Healthy Cell Bias

In 2005, Brinton [139] proposed an alternative to the critical window hypothesis, which postulated that the effect of estrogens depends on the health of a cell. As greater cell health is associated with youth, the ‘healthy cell bias’ hypothesis also supports the importance of the timing of HRT initiation, favoring the likelihood of HRT having a beneficial effect if initiated closer to the age of menopause [139,140]. A key factor that distinguishes the critical window hypothesis from the healthy cell bias is that only the latter considers baseline characteristics, such as neurological status, as an essential feature in predicting the efficacy of HRT. The results of WHIMS showed that women who had adverse effects of HRT were more likely to have lower baseline scores on the miniature mental state examination (MMSE) [23]. Furthermore, in the WHIMS-MRI study, which examined differences in brain structure, loss of brain volume (in both the frontal lobe and the hippocampal region) was more pronounced in women with low baseline MMSE scores [53]. Tierney et al. [54] also showed that in women who performed at or above a level that was expected of their demographic when assessing baseline cognition scores, HRT caused a significant enhancement of verbal memory. However, this finding was not emulated in women who did not meet this initial predicted score.

In 1999, Slooter et al. [17] demonstrated that HRT use was associated with a lower incidence of early-onset AD. As this form of AD is believed to be determined by a genetic predisposition due to the inheritance of AD-associated genes, this led to increased interest in the prevalence of genetic biomarkers of AD in study populations of clinical and epidemiological estrogen-related trials. Estrogens are known modulators of the apolipoprotein (*APOE*) gene, encoding the APOE protein, which has roles in cholesterol transport, maintenance of neuronal membranes, and amyloid distribution [141,142,143,144,145]. The presence of an ε4 allele polymorphism in exon 4 of *APOE* is associated with an increased Aβ oligomerisation and risk of AD. Screening results of the *APOE* sequence found that the promoter region, Intron 2, and Exon 4 all display high match to the consensus ERE sequence, supporting specific binding of the nuclear estrogen receptor complex [146]. Interestingly, the APOE ε4 genotype is a greater risk factor for developing AD in women compared to men, thus providing a conceivable site at which estrogens can have allelic-dependent interactions with AD, although the functional outcome of this interaction is debated [145,146].

In the MIRAGE study, favorable results of HRT on dementia outcomes were reported in a cohort where 66% of AD patients possessed an APOE ε4 allele [126]. The KEEPs study [55] also reported that HRT therapy was more effective in preventing amyloid deposition in those participants who were carriers of this allele. HRT has also been shown to slow telomeric shortening in APOE carriers [147]; furthermore, evidence from Rippon et al. [34] shows clear support for an association between HRT use and an 11-fold increase in AD diagnosis in women who were APOE ε4-positive with previous history of stroke. This research would indicate that the success of HRT is also influenced by predetermined factors such as gene status, which can exacerbate other conditions, especially in the cardiovascular system [56,148].

In addition to HRT, other AD treatments are also known to be subjected to healthy cell bias. Acetylcholine (ACh) neurotransmission is one of the significant systems affected by the pathology of AD, causing presynaptic cholinergic deficits, which result in substantial effects on cognitive decline associated with the disease [149,150]. Cholinesterase inhibitors are commonly prescribed to patients to prevent neurotransmitter breakdown and counteract this neurochemical imbalance. However, as AD progresses, the degeneration of ACh-producing cholinergic neurons becomes more widespread, and the efficacy of treatment declines as the availability of the appropriate substrate diminishes [151]. Estrogen receptors also colocalize with tropomyosin-related kinase receptor A (TrkA), tropomyosin-related kinase receptor B (TrkB), and neurotrophin receptors (NR) in basal cholinergic neurons of the forebrain (BFCN) [152]. TrkA binds to nerve growth factor (NGF) and TrkB binds to brain-derived neurotrophic factor (BDNF). This NR/growth factor system plays a critical role in the survival, differentiation, and repair of these neurons. By altering estrogen levels, NR expression also changes, with estrogen depletion resulting in rapid reductions in the number of receptors [152,153]. Ovariectomized rats’ estrogenic treatment leads to upregulation of BDNF mRNA expression in the cortex and olfactory bulbs (which are connected to BFCN through afferent innervation) and provides neurotrophic support for these neurons [154]. The increase in BDNF expression by estrogen therapy will also positively impact neurogenesis [155,156,157], an important event negatively affected by aging and disease conditions such as in AD [154,157]. This indicates that estrogens act directly on the neurotrophin system, and therefore, may be one example of a substrate that estrogen requires to have an effect.

Another well-recognized feature of neurodegenerative conditions, including AD, is neuroinflammation [158,159,160]. As such, it is important to consider the anti-inflammatory effect of estrogen as a critical and powerful factor utilized in the maintenance of normal neuronal and glial activity [161,162], a function which appears to decline with age and during menopausal transition [163]. The estrogenic stimulation of ER on glial cells acts to increase neurotrophic signaling through a mechanism likely to involve the release of TFG-β1 [164] and regulation of GFAP and glutamate transporters. Reactive gliosis is increased in young and aged animals with limited circulating estrogen levels and normalized with estrogen replacement [165].

Despite the promising evidence aligning with the critical window hypothesis, a question that remained unanswered is how estrogen treatment becomes detrimental to cognitive health. Cognitive decline and increases in the incidence of dementia imply that HRT under some conditions causes detrimental effects on the cell or neural network rather than it losing effectiveness as the sensitivity decreases. The healthy cell bias theorizes that as the cell deterioration progresses or becomes more widespread, the function of the cell, and therefore, the effect that HRT has, is also altered. This is also evidenced in in vitro models, Chen et al. [166] found that E2 treatment was most effective when administered before the induction of an Aβ insult, whereas E2 treatment, when administered after, was ineffective in reversing damage and further exacerbated the effects of the Aβ peptide.

Preceding even the prodromal stage of AD, neuronal energy production is strained as glucose utilization is impaired [167,168]. Estrogens substantially affect mitochondrial bioenergetics and cellular respiration, playing a key role in regulating calcium homeostasis and coupling glycolysis to oxidative phosphorylation [108]. In transgenic AD mouse models, estrogen depletion resulted in an energetic switch from glycolytic metabolism to ketogenic [169]. Estrogen is also reported to influence calcium dynamics, where, among other calcium-dependent neuronal processes, changes to the rapid exchange of intramitochondrial calcium affects the buffering and storage capacity of the matrix and toxic build-up of calcium in the cytoplasm [140]. E2 treatment was shown to regulate calcium levels after excitotoxicity while maintaining respiration processes [170]. It is the decreased energy production in the brain that is often a prominent underlying insult during reported neurological impairment as the demand for ATP during critical cellular events cannot be met, contributing to potentially catastrophic and irreversible changes in cell health.

More recently, estrogens have also been shown to regulate the synthesis and functionality of microRNA, small non-coding RNA molecules that act to translationally repress targeted mRNA for higher-order control and regulation of protein synthesis [171]. In animal models, E2 action on microRNA appears to act in both a tissue-specific and age-specific manner [172]; furthermore, 8 weeks of estrogen deprivation significantly affected the expression of microRNA and protein availability upon reintroduction of estrogen treatment [173]. This modulation of estrogenic activity did not appear to be related to ER availability and may contribute to detrimental effects of HRT if administered after a period of low estrogen levels. As evidenced, the temporality of estrogenic intervention appears to be essential to the efficacy of the treatment. However, as pathological disruptions in AD are antecedents of symptoms, determination of this timeline and planning intervention accordingly is difficult.

## 5. Conflicting Evidence

Thus far, the nine RCTs of AD patients have mixed results, with five studies showing no substantial differences in cognition or function [29,57,58,59,174], while the remaining four showed improvements in cognition [60,61,62,137]. Observational studies in AD patients reflect this, with HRT improving cognitive function in a wide range of neurological and neuropsychiatric tests, including olfactory odor recognition, MCI, regional blood flow and EEG activity [175,176,177]. However, Levine and Battista [63] found the opposite, showing HRT caused further decline in patient scores. Extrapolation of the findings of individual trials to form a cohesive conclusion for women’s health is difficult; however, the holistic view of all evidence does suggest that estrogenic intervention is most effective as a prophylactic option over a treatment paradigm, with the potential for preventing the delay and onset of AD symptomatology being much higher if used before neurological insults, and therefore, is a much more valuable treatment option in younger women before degenerative damage has occurred.

Despite a number of comprehensive studies that support the critical window and healthy cell hypotheses, these theories have not been unopposed. Younger, recently menopausal women have also been shown to have no change in cognitive performance [178,179], while RCTs of older women have still shown beneficial effects of HRT [32,66,180]. An RCT of 38 women who were on average 17 years post-menopause, confirmed that some tissue remains sensitive and responsive to estrogenic treatment as HRT administration correlated to increased estradiol levels and rapid improvements in cognitive performance [64]. Additionally, there are several published studies that do not show any association between HRT and outcome measures regardless of time since menopause [67,68], stage of menopausal transition [69], or by the timing of HRT initiation (The Three Cities Study, [33]; Nurses’ Health Study, [70]). The vast amount of variation in the data between studies investigating the same hypotheses indicates that other parameters within the study design have crucial and substantial effects on the outcome measures.

## 6. Additional Factors

The criteria and clinical guidelines used to recommend and prescribe HRT vary at both regional and national levels. According to the North American Menopause Society, the recommendation for women aged 60 years or younger who are within 10 years of the onset of menopause and have no other contradictions, HRT should be used; however, there are no optimal parameters of prescription that are strictly recommended, this decision often is the responsibility of the primary physician. There is accumulating evidence to support the notion that reproductive history, HRT formulation, administration route, regime, and dosage can cause differences in risk and outcomes associated with several different pathologies, and may, in part, be responsible for differences in results from clinical trials of HRT and AD, and thus, should be considered carefully.

### 6.1. Reproductive History

Determining the span of lifetime endogenous estrogenic exposure can not only be defined by age of menarche and the onset of menopause, but must also consider reproductive health, history, and personal consequences dictating parenting practices. During pregnancy and breastfeeding, the expression pattern of ovarian hormones is altered. Serum levels of E1 and E2 are drastically elevated during the first trimester of pregnancy and continue to climb until parturition. Peak levels of E1 and E2 can rise over 100-fold and 1000-fold, respectively, during gestation. Postpartum, estrogen levels drop rapidly, returning to pregravid levels within 5 days [181]. Consequently, parity has a significant impact on the total estrogen bioavailability experienced by women. Smith et al. [182] and Ragson et al. [183] aimed to define cumulative estrogen exposure more accurately to determine the risk of AD and cognitive decline. Both studies found that longer estrogen exposure was associated with improved cognitive function. More recently, Fox et al. [38] considered total lifetime duration of estrogen exposure, the number of menstrual cycles, parity, age at first birth, and age of onset of AD as variables. They found that women who spent more months of pregnancy or first gave birth after the age of 21, exhibited a lower risk of AD [38]. However, this finding is slightly controversial.

Despite greatly increased levels of estrogen during pregnancy, ovulatory cycling usually remains suppressed in anovulation for 1.5 months or 3 months for non-breastfeeding and breastfeeding women, respectively [184]. During this time, estrogen levels remain low, and it is this long-term reduction in estrogen that has led to the belief that increased parity is a risk factor for AD [185,186]. Indeed, in McLay et al. [186] nulliparous women performed better on MMSE, and in Colucci et al. [40] three or more pregnancies gave the mother a 3.2-fold increased risk of AD. A similar contradiction has been reported for breastfeeding, where longer periods of lactation have been shown to increase dementia risk and cognitive deficits [41,187], and decrease dementia risk [39].

The use of combined hormonal contraception (estrogen and progesterone) also increases cumulative estrogen exposure. Egan and Gleason [188] found evidence that any history of hormonal contraception was associated with increased performance in cognitive tests later in life; interestingly, this observation was also dependent on duration of use. Furthermore, Gong et al. [189] showed that oral contraceptive pills were protective against the risk of dementia. Clearly, reproductive factors have substantial impacts on estrogen exposure, and therefore, active reporting and consideration of these variables may provide a better indication of the value of HRT and the therapeutic efficacy.

### 6.2. Route of Administration

HRT administration can be administered via oral, transdermal (sprays, patches and gels), or vaginal courses. Although some delivery methods are preferred due to ease and higher patient compliance rates, the route of administration can have drastic effects on absorption and metabolism. Oral HRT is commonly used in clinical studies due to its ease of administration and compliance checks. Meta-analyses of estrogen preparations did not show any significant differences in efficacy between transdermal HRT and oral HRT in reducing vasomotor symptoms. However, oral administration has been associated with an increased risk of AD after long-term use; a consequence that was not observed in vaginal cream users [35]. Additionally, in ‘Estrogen and Thromboembolism Risk Study’ (ESTHER, [190]), the risk of venous thromboembolism was associated with oral but not transdermal estrogen delivery, indicating a clinically relevant difference in the estrogenic effect between these delivery routes [190]. As transdermal and vaginal delivery systems can bypass the ‘first pass’ of the hepatic metabolism, these routes are able to maintain more of the drug’s bioavailability and correlate to higher serum levels at lower doses compared to oral prescriptions. Differences in safety may also be accounted for by the impact of hepatic protein synthesis and clearance. Deleterious byproducts are produced or are subject to reduced clearance, a process that can also vary further depending on liver health and, as in older age, impairments to liver function can be a significant factor that needs to be accounted for when choosing which route of administration should be used.

### 6.3. Formulation

The most commonly prescribed HRT in the United States is conjugated equine estrogens (CEE), which contain prodrugs of E1 and equilin, an estrogenic sex hormone found in horses [191]. CEE has been shown to have detrimental effects in American studies: Maki [176], Shaywitz et al. [71], Ditkoff et al. [192], WHI/WHIMS and corresponding ancillary studies. While in European studies with similar parameters (including population demographics, outcome measures, and duration of treatment) the favored use of E2, a lone estrogenic formula demonstrated a beneficial effect of E2 such as in Sherwin [193], Sherwin [72], Phillips and Sherwin [73] and Imtiaz et al. [27].

To date, no direct comparison of E2 and CEE on dementia outcomes has been attempted in a clinical setting; however, in the KEEPS study, transdermal CEE showed higher levels of Aβ deposition than users of transdermal E2 users [55]. Furthermore, epidemiological analysis of AD patients has shown that E2 use was of greater benefit to verbal memory than CEE [74,75]. Chang et al. [194] demonstrated similar findings, showing that the action of CEEs caused a 1.17-fold increase in the incidence of ischemic stroke compared to users of E2. These results are likely dictated by differences in each formulation’s opportunity for conversion and binding affinity.

Unlike E2 levels, serum levels of E1 are resistant to change during the menopausal transition and remain relatively stable [195]. Consequently, the body does not have the same deprivation of E1 as E2 right after menopause. ERs in the brain also have a greater affinity for E2 than CEE [196]. Although E1 can be converted into E2 through a 17β- hydroxysteroid dehydrogenases-dependent process, E2 also provides greater serum levels of E2 than CEE when using the same dose, indicating that complete conversion cannot be achieved [197,198,199]. The enzymes required in the metabolism of E1 are also known to become dysfunctional in AD [200,201]. Equilin can also be converted to a more potent ER activator (17β-dihydroequilin); however, the signaling profile and the ability to mediate neuroprotective effects have not been well studied. Additional evidence shows that E2 improves cognitive function more effectively than CEE due to differential actions on proteins and enzymes in addition to ERs [202].

### 6.4. Regime and Dosage

The optimal dosage of estrogen as a replacement therapy should aim to use the lowest effective dose to supplement E2 levels back to what was found during an individual’s premenopausal physiological conditions. The estrogenic environment in premenopausal women fluctuates with the menstrual cycle resulting in sampled serum estradiol levels ranging between 30 and 800 pg/mL, peaking during the midfollicular phase and falling after ovulation [203]. The regime and dose of estrogen have been tested in in vitro studies to maximize the neuroprotective effect. The three most documented HRT regimens include acute, continuous and cyclic/intermittent dosing, the last of which was developed to mimic the natural premenopausal cycle. In Chen et al. [166], low E2 (10 ng/mL) and high E2 (200 ng/mL) were evaluated in rats using all three temporal regimes. The effect on amyloid-induced neurodegeneration in hippocampal neurons between doses was apparent, with low E2 proving to be beneficial in all exposure patterns, while high E2 resulted in increased Aß^1-42^-induced neurodegeneration.

In humans, standard oral doses for CEE and E2 are 0.625 mg/day and 2 mg/d, respectively. Standard (0.625 mg/day) and high dose oral CEE (1.25 mg/day) both had detrimental effects on the Clinical Dementia Rating Scale, while only the lower dose had a beneficial impact on the MMSE [57]. The use of transdermal E2 (0.10 mg/day) for 8 weeks showed marked improvements in cognitive neuropsychometric tests compared to placebo [61], while a higher dose (0.25 mg/day, [68]), and ultra-low dose transdermal E2 (0.014 mg/day, [45]) had no effect on change in cognitive function over the 2-year follow-up period. While further investigation comparing different doses in the same trial would offer more conclusive evidence, the therapeutic window appears to be narrow, and therefore, dose titration may be an important component in managing the risks of prescribing HRT.

The duration of treatment also appears to influence the cognitive outcome of HRT users. In the Kaiser Permanente study, unlike the reported incidence of short-term midlife users for whom HRT had a beneficial effect on dementia, for late-life users there was a marked increase in risk. In long-term HRT users (at both mid- and late-life) no change in AD incidence was found compared to never users. These results could be interpreted as the beneficial effects of estrogens when used in mid-life were ‘neutralized’ by the detrimental effects of HRT in later life. Interestingly, this ‘turning point’ of estrogen’s effects is also seen in the treatment of other chronic diseases such as atherosclerosis and osteoporosis [204]. Yoo et al. [25] also showed that while the incidence of AD was still decreased from non-users, the incidence of cases increased with the duration of HRT use (in years). Additionally, the validity of some clinical studies that have very short intervention periods, such as Wolf et al. [64] that drew conclusions on HRT after only 2 weeks, should be considered very carefully, as it is possible that the duration of the HRT intervention was not long enough to produce a true effect on cognition or dementia risks.

### 6.5. Progestrone (Combined Therapy)

Primarily responsible for supporting pregnancy, progesterone’s hormonal action opposes estrogen, ultimately preventing abnormal growths in the endometrium and ovaries to decrease cancer risk [205]. As HRT is associated with increased risks of breast and uterine cancers, it is often a combination therapy, with co-administered progesterone (commonly: medroxyprogesterone acetate (MPA/Progestin), norethindrone acetate (NETA), or micronized progesterone (MP)) in women with intact uteruses that successfully mitigates this risk [206]. Progesterone also has its own neuroprotective activity, regulating synaptogenesis and neuronal plasticity through cell-survival-signaling pathways; however, when administered in combination with estrogen, this treatment has been well documented to have net adverse effects on cell health [207,208,209,210,211].

The results from the LAW study and WHI-WHIMS strongly favored the use of estrogen-only treatments in decreasing AD incidence [24,28]. Additionally, in two randomized control trials, the Women’s Health Initiative Study of Cognitive Aging (WHISCA, [42]) and the Heart and Estrogen/progestin Replacement Study (HERS, [76]) the outcome of CEE was compared with and without MPA. In both studies, the combined group worsened cognition compared to CEE alone. These detrimental outcomes may be due to the antagonizing effect of progesterone on estrogen receptors, modulating the influence of estrogen, and neutralizing neuroprotective effects [111,170,212]. Despite recognizing and acknowledging the consequence combination therapy has on HRTs beneficial effects regarding cognition, cancer risk complications are also a serious concern and should not be overlooked.

## 7. Selective Estrogen Receptor Modulators and Activators of Non-Genomic Estrogen-like Signaling

As estrogens can cross the blood–brain barrier, HRT has the valuable quality of being able to act on both the peripheral organs and the central nervous system. This allows HRT to be able to modulate menopausal symptoms in addition to having an effect on cognitive domains and processes in the brain. However, considering collateral damage produced as a result of HRT, treating one system at the cost of another is a serious factor when recommending HRT for conditions such as AD. A new body of literature on synthetic neuro-steroids has arisen from the identification and engineering of new compounds, which are structurally distinct from estrogens but capable of selectively activating estrogen-signaling mechanisms. The advantage of these compounds, which mimic estrogenic effects, is that they may be able to circumvent the detrimental effects of HRT while still offering some of the beneficial effects- improving the safety profile and tolerability of HRT.

Selective estrogen receptor modulators (SERM) have tissue-type-specific action, with both estrogenic and antiestrogenic actions. This quality of SERMS allows greater control over the differential effect of estrogens across the body. Two SERMS, tamoxifen (TMX), and raloxifene, have recently been discussed with respect to their potential to provide neuroprotection [213]. The pharmacodynamic effects of SERMS categorize TMX and RXF as partial agonists of ERs. TMX has differential actions on genomic estrogen-signaling mechanisms, antagonizing ERE sites but having agonistic effects on AP-1, although this selective action is not well understood. In the ‘Multiple Outcomes of Raloxifene Evaluation Trial’ [214], postmenopausal women showed a dose–response relationship, with higher doses of RXF (120 mg) resulting in a lower incidence of AD and improved mild cognitive impairment in the preclinical stages of the disease. However, high doses of SERMS have also been associated with retinopathy, cataracts, increased risk of thromboembolic events, and breast cancer due to their estrogenic effect on bone, liver, and blood. Recently, research has shifted to investigating the combination treatment of estrogens and SERMs known as a tissue-selective estrogen complex (TSEC) to better affect the spectrum of postmenopausal symptoms while maintaining an estrogenic effect in the brain. Despite being a relatively novel treatment, TSECs have increased tolerability in patients and are still effective in reducing menopausal symptoms [215].

Other alternatives to HRT include selective tissue estrogenic activity regulators (STEARS) [216], and selective estrogen receptor degraders (SERD), which have had some minor successes in demonstrating therapeutic effects. Structurally different from SERMS, STEARS, such as tibolone (TIB), can have progestogenic effects in endometrial tissue, estrogenic effects in bone and vaginal tissue, and androgenic effects in the liver and brain, which may provide some neuroprotective effects [217,218]. Although, like conventional HRT, TIB has also been the subject of conflicting reports [35,219]. Alternately, the SERD, fulvestrand, is a pure estrogen receptor antagonist that can down-regulate receptor activity in addition to blocking, which could be tactically employed to reduce overstimulation of specific ERs to reduce unwanted effects [220]. While improvements to the molecular structures of these molecules are needed to have a more substantial and useful effect, designer compounds, such as STEARS and SERD, could have promising capacities to increase the specificity of the estrogen impact in the body.

More recently, three ‘activators of nongenomic estrogen-like signaling’ (ANGELS), including estren, compound A and compound B have received significant attention. Estren, in particular, has been demonstrated to protect agonist Aβ pathology and restore cholinergic neuron density to a similar extent as E2, in addition to having restorative actions of bone loss and cholinergic neurons without affecting reproductive tissues [221,222,223,224]. However, the proposed mechanism of action of ANGELS is not well understood, and it has been hypothesized to involve imperfect and transient binding to membrane ERs. ANGELS activate non-classical signaling through ERα and mitogen-activated protein kinase (MAPK) and ERK activation [223]. ANGELS could also have an effect on treating menopausal symptoms, and their development is an exciting step toward creating neuroprotective drugs for use against multiple estrogen-sensitive brain pathologies, without sacrificing reproductive health [222].

There is also some evidence to suggest that phytoestrogens could be beneficial in postmenopausal women. Phytoestrogens are plant-derived estrogens with a similar chemical structure and binding ability to human ERβ [225]. Genistein and s-equol, two phytoestrogens, have also been shown to protect DNA and normal mitochondrial functions, as well as increase the synthesis of antioxidant and anti-inflammatory molecules. Supplementing these micronutrients by consuming foods high in phytoestrogens, such as soy, berries, cereals, and beans, has been explored as a potential method for neuromodulation. Furthermore, increased endogenous levels of estrogens have been shown to correspond to an apparent strengthening in the protective effects of exogenously administered steroids [226,227]. Therefore, a diet that supports the maintenance of E2 levels for longer could play a role in the promotion of cognitive health in peri- and postmenopausal women, but more research is required to assess these positive effects of phytoestrogens that might support dietary recommendations. This research would also inform us about the possible negative effects of phytoestrogen consumption, such as that on fertility and birthing [228], obesity [229,230,231], and breast cancer [232].

## 8. Conclusions

Estrogens have several complex mechanisms of action through which hormones can exert valuable effects on cellular functioning. Demonstrating the effects of HRT on preserving neuronal health through estrogens ameliorative actions, resulting in beneficial effects on cognitive health and the incidence of dementia, has significant implications for the future of women’s health. However, these effects appear to be tightly governed by several regulating factors and mechanisms that are not yet well understood. This has resulted in greatly dispersed and conflicting evidence regarding the therapeutic benefit of HRT. It is apparent that both known and unknown factors have contributed to the outcome of studies that were unsuccessful in showing a beneficial effect of HRT, although the extent to which they have interfered with results is unknown. A major challenge for future research will be to attempt to isolate the many factors that appear to contribute to the efficacy and vector of estrogenic intervention to aid attempts to establish a reliable treatment protocol of HRT that is protective against cognitive decline.

Research on neurological health in women has been neglected for decades despite crucial information that could be learned from advancing research in this area. Estrogens play a clear role in modulating the risk of dementia, with reliable evidence showing that HRT can have a beneficial and deleterious effect. Investigating the molecular targets, pathways, and neurological systems along which estrogens interact to facilitate this spectrum of consequences will aid our understanding of the mechanisms involved in cognition and the numerous factors that contribute to the onset and development of AD. HRT appears to be a potent and effective therapeutic option for protecting against AD in young women. However, the recommendation for HRT use should consider baseline characteristics such as genotype, cardiovascular health, and education, as well as age, dose, and duration of treatment until the risk factors that modulate the effects of HRT can be investigated more thoroughly or improvements in alternative treatments can be made.

## Figures and Tables

**Table 1:** Summary of human observational and clinical studies included in this review.

Authors (Year)	Origin	Study Type	Study Groups	Outcome	Type	Dose (mg/day)	ROA	Menopause Stage	Age (Years)	Duration of HRT Use	Main Finding or Conclusion (HRT Related)
Mortel and Meyer (1995) [13]	USA	Observational	Control HRT− (*n* = 75) Control HRT+ (*n* = 17) AD HRT− (*n* = 158) AD HRT+ (*n* = 10)	HRT use and probable AD diagnosis	NR	NR	NR	Postmenopausal	>60	NR	Non-patients were more likely to have taken HRT than patients diagnosed with AD. This evidence suggests that HRT may be a prophylactic agent in reducing dementia of the AD type in postmenopausal women.
Tang et al. (1996) [14]	USA	Observational	Control HRT− (119) Control HRT+ (29) AD HRT− (86) AD HRT+ (7)	HRT use and AD diagnosis	CEE Other	NR	NR	Postmenopausal	Mean 74.2 ± 7	≤1 >1	The annual incidence of AD was lower among users of HRT. Long-term HRT use over 1 year provided the lowest risk for AD.
Kawas et al. (1997) [15]	USA	Observational	Control HRT− (810) Control HRT+ (147) AD HRT− (158) AD HRT+ (9)	HRT use and AD diagnosis	NR	NR	Oral TD	Postmenopausal	28–94 Mean 61.5	<0–5 years 5–10 years >10 years	Fewer diagnoses of AD were made among women taking HRT. HRT showed a protective influence against AD, with no effect associated with the duration of HRT use.
Baldereschi et al. (1998) [16]	ITA	Observational	Control HRT− (217) Control HRT+ (221) AD HRT− (25) AD HRT+ (9)	HRT use AD diagnosis Cognitive tests: MMSE, CAMDEX, Pfeffer Functional Activities Questionnaire, HDRS.	NR	NR	NR	Postmenopausal	65–84	NR	There was a decreased risk of AD among women taking HRT. The age of disease onset did not differ between groups. Women taking HRT had better scores on the MMSE test.
Slooter et al. (1999) [17]	NLD	Observational	Control HRT− (1293) Control HRT+ (183) AD HRT− (89) AD HRT+ (3)	HRT use and AD diagnosis	NR	NR	NR	Postmenopausal	<65	NR	HRT use was negatively associated with the development of early onset AD.
Waring et al. (1999) [18]	USA	Observational	Control HRT− (95) Control HRT+ (24) AD HRT− (98) AD HRT+ (11)	HRT use and AD diagnosis	NR	NR	Oral Topical	Postmenopausal	57–96	<6 months >6 months	HRT use was more common among non-patients than AD patients. Increased duration of HRT use positively correlated with decreased AD risk.
Lindsay et al. (2002) [19]	CAN	Observational	Control HRT− (175) Control HRT+ (47) AD HRT− (189) AD HRT+ (33)	HRT use and AD diagnosis	NR	NR	NR	Postmenopausal	65+	NR	HRT was not a protective or adverse factor for the risk of AD.
Zandi et al. (2002) [20]	USA	Observational	Control HRT− (1837) Control HRT+ (125) AD HRT− (106) AD HRT+ (4)	HRT use and AD diagnosis	NR	NR	NR	Postmenopausal	Mean 74.5	<3 years 3–10 years >10 years	Previous HRT use was associated with a reduced risk of AD. This benefit was not observed if HRT was used for a duration of more than 10 years.
Yoo et al. (2020) [25]	KOR	Observational	HRT− (3,641,065) HRT+ (679,456) AD HRT− (189,459) AD HRT+ (14,151)	Dementia diagnosis AD diagnosis Cognitive tests: MMSE	NR	NR	NR	Postmenopausal	Mean 61.2 ± 8.6	Mean 5.74 years	The risk of dementia increased in women who reached menarcheal age at an older age, and decreased for women who reached menopausal age later. The use of HRT reduced the risk of dementia. The findings from this study demonstrate that female reproductive factors are risk factors for dementia incidence with higher risk associated with shorter lifetime endogenous estrogen exposure.
Shao et al. (2012) [26]	USA	Observational	Control HRT− (574) Control HRT+ (1081) AD HRT− (89) AD HRT+ (87)	HRT use AD diagnosis	Unopposed Opposed Unknown	NR	Oral TD	Postmenopausal	65+	<3 years 3–10 years ≥10 years	Women who began HRT within 5 years of menopause showed a decreased risk of AD. This risk was shown to decrease further if HRT was used for 10 years or more. This effect was not seen in women who initiated HRT 5 years or more after menopause. Additionally, unopposed HRT use, but not opposed HRT use, was associated with a decreased AD risk.
Imtiaz et al. (2017) [27]	FIN	Observational	Whole cohort (8938)	AD diagnosis	Unopposed Opposed	NR	Oral TD	Postmenopausal	47–56	<1 year 1–3 years 3–5 years 5–10 years >10 years Follow up: 20 years	There was no association found between HRT use and AD risk. In long-term users of unopposed HRT, self-reported data showed a decreased risk of AD although there was no evidence to suggest a protective association.
Imtiaz et al. (2017) Tuppurainen [27]	FIN	Observational	Whole cohort (8195)	AD diagnosis	Unopposed Opposed	NR	Oral TD	Premenopausal Menopausal Postmenopausal	47–56	<1 year 1–3 years 3–5 years 5–10 years >10 years Follow up: 20 years	A protective association was found between postmenopausal HRT use and AD.
Imtiaz et al. (2017) [27]	FIN	Observational	HRT− (488) HRT+ (243)	MCI diagnosis Dementia diagnosis Cognitive tests: MMSE. Immediate word recall, Stroop Test, CF Test, Bimanual Purdue Pegboard Test, LD substitution test.	Unopposed Opposed	NR	NR	Postmenopausal	65–79	<5 years <5 years Follow up: 8 years	Overall, no strong protective effect of HRT on cognition was found. Long-term HRT users had improved global cognition in some domains.
Khoo et al. (2010) [28]	AUS	Observational	HRT− (213) Early HRT+ (158) Late HRT+ (39)	Cognitive test: MMSE, NART, WMS-3, General Memory Index, Working Memory Index.	Unopposed Opposed	NR	NR	Postmenopausal	40–80	>12 months Follow up: 5 years	Early start use of unopposed HRT was associated with a reduced risk of global cognitive decline, while opposed HRT showed increased risk of memory decline. There were no major effects on subgroup regarding type or timing of HRT on cognitive function. This study suggest that cognitive effects of hormone therapies may be mixed depending on cognitive domain, timing of use and preparation of HRT.
Henderson et al. (2005) [29]	USA	Observational	Control HRT− (353) Control HRT+ (192) AD HRT− (339) AD HRT+ (87)	HRT use AD diagnosis	NR	NR	NR	Postmenopausal	65+	>6 months	The findings of this study support evidence of a critical window of initiation for HRT use and AD risk. Association of HT use and AD risk was found to be dependent on timing of use with a significant protective association found only in the youngest age tertile.
Greendale et al. (2009) [30]	USA	Observational	Control HRT− (1887) Pre/Peri HRT+ (380) Post HRT+ (95)	Cognitive tests: SDMT, EBMT, DSB.	NR	NR	NR	Peri-menopausal Post-menopausal	42–59 Mean 45.9	2- 3.2 years prior to MP Follow up: 4 years	HRT initiated pre-menopause was shown to have a beneficial effect on cognitive performance, while initiation post-menopause was detrimental. There was no difference in performance scores between postmenopausal non-HRT users and that of premenopausal women.
MacLennan et al. (2006) [31]	AUD	Observational	Never Users (194) Current HRT Users (70) Past HRT Users (140)	Cognitive Tests: MMSE, CERAD, VFT (FAS), BNT, TMTA and TMTB.	Unopposed Opposed	NR	NR	Postmenopausal	60+	<1 year 1–5 years <6–11 years >11 years	Early initiators of HRT performed better on cognitive tests than never users. Late initiators performed worse than never users on the MMSE but not the FAS test. Early initiation of HRT showed beneficial effects, while later initiation may have a detrimental effect on cognitive performance.
Slooter et al. (1999) [17]	NLD	Observational	Control HRT+ (24) Control HRT− (95) AD HRT+ (11) AD HRT− (98)	AD diagnosis	NR	NR	NR	Postmenopausal	AD onset <65	NR	An inverse association was found between early HRT use and early onset AD, where HRT was beneficial in risk reduction.
O’Hara et al. (2005) [32]	USA	Observational	HRT+ (37) HRT− (32)	Cognitive tests: MMSE, Revised Benton Visual Retention test, WMT, symbol digit modalities test, list learning measure of delayed recall, GDS	Estradiol Valerate ± Norethisterone	0.625 ± 2.5	Oral	Postmenopausal	60–93 Mean 72.74 ± 7.58	5 years	Findings from this study showed no effect on HRT on any measures of cognition.
Ryan et al. (2009) [33]	FRA	Observational	Never Users (2169) Past HRT+ (487) Current HRT+ (474)	Dementia diagnosis Cognitive tests: MMSE, Issacs Set Test, Benton Visual Retention Test, immediate and delayed recall (word recall) Trail Making Test A and B.	17-β Estradiol	NR	NR	Postmenopausal	Never 74.9 ± 5.3 Past 72.4 ± 4.7 Current 70.2 ± 3.3	4 years	Current HRT users had greater performance on verbal fluency, working memory and psychomotor speed compared to never users. The strength of association varied depending on the type of treatment with longer duration appearing to be more beneficial. However, initiation of HRT close to menopause was not associated with improved cognition. HRT did not significantly reduce the risk of dementia.
Rippon et al. (2006) [34]	USA	Observational	HRT+ (77) HRT− (289) AD HRT+ (19) AD HRT− (61)	AD diagnosis	NR	NR	NR	Postmenopausal	HRT+ 63.97 ± 9.98) HRT- 74.4 ± 13.4	NR	In women with history of stroke, ERT was a deleterious effect monitor.
Savolainen-Peltonen et al. (2019) [35]	FIN	Observational	Control HRT− (59,175) Control HRT+ (25,564) AD HRT− (58,186) AD HRT+ (26,553)	HRT use AD diagnosis	Unopposed Opposed Tibolone	NR	Oral TD Vaginal	Postmenopausal	50+	<3 years 3–4 years 5–9 years ≥10 years	Long-term use (10+ years) of HRT was associated with an increased risk of AD, regardless of use of opposed or unopposed HRT. This increased risk remained apparent after 3–5 years of HRT use in older women. Users of vaginal estradiol did not show the same increased risk.
Matyi et al. (2019) [36]	USA	Observational	Whole cohort (2114)	Dementia screening Cognitive tests: 3 MS	Unopposed Opposed	NR	NR	Postmenopausal	74.94 (mean age)	HRT duration was used as a variable	Longer duration of HRT exposure was associated with better cognitive health. Women who initiated HRT earlier showed higher cognitive scores than those who initiated later.
Kim et al. (2022) [25]	KOR	Observational	Dementa—(183,510) Dementia + (26078)	Dementia diagnosis (AD and vascular)	NR	NR	NR	Postmenopausal	Dementia—(61.54) Demenia + (70.46)	<2 years 2–5 years >5 years Unknown Follow up: 7.72 years	HRT use after menopause transition was associated with decreased risk of AD and VD in depressed female patients. Lifetime oral contraceptive use was also associated with lower AD risk.
Lokkegaard et al. (2022) [37]	NLD	Observational	Dementia + (2235) Dementia—(11028) Dementia twin + (204) Dementia twin—(204)	HRT initiation (age) Dementia diagnosis (age)	Unopposed Opposed Continuous Cyclic	NR	Oral TD	NR	45+ (for first HRT use)	NR	HRT use was associated with increased dementia risk if used before 2003.
Fox et al. (2013) [38,39]	ENG	Observational	AD (38) Controls (51)	Endogenous estrogen exposure span Reproductive factors HRT use	NR	NR	NR	NR	70 -100	NR	HRT had no effect on AD risk. Increased total months of endogenous estrogen exposure did result in a negative associated risk of AD.
Colucci et al. (2006) [40]	ITA	Observational	AD HRT− (188) AD HRT+ (16) Control HRT− (167) Control HRT+ (34)	HRT use Reproductive factors	NR	NR	NR	NR	AD (75.3) Controls (74.3)	NR	The AD cohort had a higher number of pregnancies than the control group. Findings suggest that is it an increase in progesterone or estrogen levels and not a decrease that is associated with AD pathology.
Hesson et al. (2012) [41]	CAN	Observational	Whole cohort (50)	Cognitive tests: STW Test, GDS, WMS-III LM I and II, time-based memory tests, RAND health survey. Estrogen exposure HRT use	NR	NR	NR	NR	69.3 (mean)	Initiated HRT within 5 years of menopause Current Former	Cumulative lifetime exposure to estrogen was associated with memory performance.
Espeland et al. (2004) [42]	USA	Clinical	Placebo− (1421) Placebo MPA− (2213) CEE+ (1387) CEE+ MPA+ (2131)	Cognitive test: 3 MSE	CEE ± MPA	0.625 mg	Oral TD	Postmenopausal	65–79	Mean 5.4 years	HRT was shown to have adverse effects on cognition. In women with lower baseline cognitive scores this effect was stronger.
Schiff et al. (2005) [43]	UK	Clinical	12 women (crossover design)	Cognitive test: simple and choice reaction time, immediate word recall, delayed word recall, digital vigilance, visual tracking, spatial working memory, word recognition, picture and face recognition, NART.	17-β Estradiol	50 ug	TD	Surgically menopausal	62–89	8 weeks follow up: for 26 weeks	HRT therapy, when administered short term, did consistently improve the speed and accuracy of older women in cognitive tests, including reaction time and depression scores. These findings support an effect of estradiol on mood and cognitive function and not simply as a result of relief of climacteric symptoms.
Almeida et al. (2006) [44]	AUS	Clinical	HRT+ (58) Placebo (57)	Cognitive tests: CAMCOG, VFT, BD, CVLT-II	17-β Estradiol	2	Oral	Postmenopausal	70+	20 weeks	High-dose HRT when administered for a course of 20 weeks was not associated with significant changes in cognitive function, mood or quality of life.
Yaffe et al. (2006) [45]	USA	Clinical	HRT+ (208) Placebo (209)	Cognitive tests: MMSE, Episodic Memory, M-BNT, Verbal Fluency, Medical Outcomes Study Health survey (SF-36)	17-β Estradiol	0.014	TD	Postmenopausal	60–80	2 years	Ultra-low dose HRT treatment for a course of 2 years had no effect on change in cognitive function of health-related life quality.
Shaywhitz et al. (2003) [46]	USA	Clinical	60 (Cross-over design)	Cognitive tests: Grey Oral reading test (3rd edition), WMS, sentence span)	CEE	1.25	Oral	Postmenopausal	32.8–64.9 Mean 51.2 ± 5	3 weeks	Participants in the HRT-active treatment arm showed better performance on oral reading and verbal memory than the placebo group. HRT had greater beneficial effects on midlife postmenopausal women.
Espeland et al. (2013) [47]	USA	Clinical	HRT+ (609) Placebo (559)	Cognitive tests: TISC-m, E-BMT, OTMT, VF-A, WAIS-R	CEE ± MPA	0.625 ± 2.5	Oral	Postmenopausal	50–55	7.2 years	HRT produced no overall sustained benefit or risk to cognitive function in any domain. HRT therapy may adversely affect verbal fluency among women with prior use of HRT.
Espeland et al. (2017) [48]	USA	Clinical	Younger placebo (635) Younger HRT+ (701) Older placebo (1478) Older HRT+ (1402)	Cognitive tests: TICS-m, E-BMT, OTMT, VF-A, DST	CEE ± MPA	0.625 ± 2.5	Oral	Postmenopausal	50–54 65–79	CEE + MPA mean 5.4 years CEE mean 7.1 years	HRT in younger women had no significant long-term effects on cognitive function or changes in cognitive function. In older women, HRT use was associated with long-term detrimental effects in global cognitive function, working memory and executive function. When HRT was initiated near the time of menopause, there was neither a benefit nor a detrimental effect on cognition.
Girard et al. (2017) [49]	FRA	Clinical	HRT+ (6) Placebo (6)	fMRI Cognitive tests: Task switching	17-β Estradiol ± Progesterone	2 100		Postmenopausal	48–55	56 days	HRT enhanced brain activation and recruitment of dorsolateral prefrontal cortex neurons during task switching, and this change was associated with the performance on the task. The results suggest that HRT, when taken early, may have a beneficial effect on cognitive control of prefrontal mechanisms.
Dunkin et al. (2005a) [50]	USA	Clinical	HRT+ (8) Placebo (9)	Cognitive tests: Stroop Task, WAIS-R, DST, Grooved Pegboard Test, CVLT, WMS-R, WCST, Controlled Oral Word Association Test, Trail Making Test—B	17-β Estradiol	0.1	TD	Postmenopausal	Control Mean: 57 ± 6.89 Depressed Mean: 54.10 ± 6.51	10 weeks	Reproductive events and levels of endogenous estrogens were found to be related to the clinical response to HRT. Overall, little beneficial effect of HRT was found although years after menopause were significantly related to change in executive functioning in the treatment group but not in the placebo. Women who were more recently menopausal demonstrated a greater positive change than older women.
Song et al. (2020) [51]	SGP	Clinical	MCI HRT− (6216) MCI HRT–(1273) HRT+ (674) HRT+ (59)	MCI diagnosis Cognitive tests: SM-MMSE	Unopposed Opposed	NR	NR	Postmenopausal	45–74 Mean 53.4 ± 6.4	NR	Users of HRT showed a decreased risk of mild cognitive impairment diagnosis compared to non-users of HRT.
Dunkin et al. (2005b) [52]	USA	Clinical	Control HRT+ (8) Control HRT− (9) Depressed HRT+ (10) Depressed HRT− (10)	Cognitive tests: Stroop Task A and B, WAIS-R, Grooved Pegboard Test, CVLT, WMS-R, WCST, Controlled Oral Word Association Test (FAS), Trail Making Test Part B, WAIS-R, AMNART, HAM-D	17-β Estradiol	0.1	TD	Postmenopausal	Control: 57 ± 6.89 Depressed: 54.1 ± 6.51	10 weeks	Recently menopausal women demonstrated greater positive change than older women, especially in domains of executive functioning. This finding occurred in both depressed and control subject.
Resnick et al. (2009) [53]	USA	Clinical	HRT+ (257) HRT+ MPA+ (435) Placebo (263) Placebo MPA− (447)	MRI Cognitive tests: 3MSE	CEE ± MPA	0.625 ± 2.5	Oral	Postmenopausal	71–89	Mean 4 years	Opposed or unopposed HRT was associated with greater brain atrophy in women ages 65+. Greater adverse effects were observed in women who had lower cognitive baseline scores.
Tierney et al. (2009) [54]	CAN	Clinical	HRT+ (70) Placebo (72)	Cognitive tests: CVLT, Rey–Osterrieth Complex Figure Test, BNT, Trail Making Test Parts A and B	17-β Estradiol + progesterone	1 1 + 0.35	Oral	Postmenopausal	61–87 HRT+ Mean 75 ± 6.4 Placebo Mean 74.5 ± 7.4	2 years	Women in the treatment group who scored above average at baseline showed significantly less decline than the placebo group in short delay verbal recall. No treatment effect was found in women who scored below average on these measures. These findings support the hypothesis that HRT will have a greater beneficial effect in women with healthy neurons.
Kantarci et al. (2016) [55]	USA	Clinical	CEE+ (29) 17 B Estradiol+ (30) Placebo (36)	MRI Cognitive tests	17-β Estradiol CEE Progesterone	50 ug 0.45 200	TD Oral	Postmenopausal	52–54	4 years	Ventricular volumes increased without changes in cognitive performance in women who received CEE compared to placebo. Rates of ventricular expansion were not different between groups; however, only differences in the CEE group reached significance.
Shumaker et al.(2004) [56]	USA	Clinical	HRT+ (1464) HRT+ MPA+ (3693) Placebo (1483) Placebo MPA− (3786)	AD diagnosis MCI diagnosis Cognitive tests: 3MSE, CERAD	CEE ± MPA	0.635 ± 2.5	Oral	Postmenopausal	65–79	Opposed: 7 years Unopposed: 9 years	Unopposed HRT did not reduce the incidence of dementia or MCI. Pooling data from unopposed and opposed groups resulted in increased risk for both dementia and MCI.
Henderson et al. (2000) [29]	USA	Clinical	AD HRT+ (21) AD Placebo (21)	AD diagnosis Cognitive tests: ADAS-cog, CGIC, ADL/IADL, MADRS, WMS, BNT, TMT, logical and visual reproduction subsets, Token Test, GDS	CEE	1.25	Oral	Postmenopausal	HRT+ 77 ± 1.4 Placebo 78 ± 1	16 weeks	Short-term HRT use did not improve global, cognitive, or functional performance among women with mild to moderate AD.
Mulnard et al. (2000) [57]	USA	Clinical	Low Dose HRT+ (42) High Dose HRT+ (39) Placebo (39)	HRT use and AD diagnosis Cognitive tests: CGIC, MMSE, CDRS, HDRS, MAACL, ADAS-cog, TMT, EFRT, BDRS, New Dot, Letter Cancellation, Digit Symbol, Category and Letter Fluency, Grooved Pegboard, Finger Tapping, Dependency Scale.	CEE	Low (0.625) High (1.25)	Oral	Surgically Menopausal	>60	48 weeks	HRT did not slow disease progression or improve global, cognitive, or functional outcomes regardless of dose. HRT group showed some benefit at 8 weeks compared to placebo, but this effect changed to become detrimental following 12 months. The CEE treatment group showed benefit at 8 weeks, became worse than placebo following 12 months of CEE.
Wang et al. (2000) [58]	TWN	Clinical	HRT+ (24) Placebo (39)	AD diagnosis Cognitive tests: CASI, MMSE, CIBIC-plus, BEHAVE-AD, HDRS, HARS, rCBF	CEE	1.25	Oral	Postmenopausal	>/=60	12 weeks	HRT did not cause any effect on tested domains in postmenopausal women with AD.
Valen-Sendstad et al. (2010) [59]	NOR	Clinical	HRT+ (33) Placebo (32)	AD Diagnosis Cognitive tests: WLM, CERAD-modified MMSE, HDRS, TMT, CERAD-modified BNT, CERAD depression screen, WAIS, Constructional Praxis, Global Deterioration Scale, Barthel Index	17-β Estradiol Norethisterone	1 0.5	Oral	Postmenopausal	65–89	48 weeks	HRT improved mood, depressive symptom scores, and appeared to slow the progression of cognitive decline. This effect was stronger in those without the APOE allele.
Asthana et al. (1999) [60]	USA	Clinical	AD HRT+ (6) AD Placebo (6)	HRT use and AD diagnosis Cognitive tests: BSRT, SCWIT, TMT, MMSE, BMICT, BPRS, paragraph recall, verbal reproduction and fluency, Token Test	17-β Estradiol	0.05	TD	Postmenopausal	HRT+ 79.5 ± 7.6 Placebo 77.6 ± 6.6	8 weeks	The HRT group showed improvement in attentional and verbal memory domains in women with AD. These improvements were not stable and did not remain after HRT discontinuation.
Asthana et al. (2001) [61]	USA	Clinical	HRT+ (10) Placebo (10)	AD diagnosis Cognitive tests: MMSE, BMICT, BPRS, CIBIC, IADL, PSMS, SCWIT, TMT, BSRT, OMDR, BNT, RCFT, Story recall, Visual Paired- Associates, Treisman VS	17-β Estradiol	0.10	TD	Postmenopausal	HRT+ 79.0 ± 9.7 Placebo 80.2 ± 6.7	8 weeks	HRT improved attention, verbal memory and visual memory in postmenopausal women with AD.
Wharton et al. (2011) [62]	USA	Clinical	Low Dose HRT+(10) Low Dose HRT+(8) High Dose HRT+ (8) High Dose HRT (9) Placebo (8)	AD Diagnosis Cognitive tests: BNT, POMS, VPA, TMT, SCWIT, List Learning, paragraph recall, Complex Figure and Figural Memory Tests.	17-β Estradiol ± MPA	0.05 ± 2.5	TD	Postmenopausal	55–85	48 Weeks	HRT administration improved semantic and episodic visual memory domains in postmenopausal women with AD. Changes to episodic visual memory were more pronounced in women receiving opposed HRT.
Levine and Battista (2004) [63]	USA	Clinical	HRT− (99) HRT+ (20)	AD diagnosis Cognitive tests: BDRS, MMSE, NCSE, CSDD, NRS, GDS	NR	NR	NR	Postmenopausal	HRT- 78.78 ± 6.38 HRT+ 77.48 ± 5.08	NR	No differences in cognition, mood and age of onset AD between groups. Women in the HRT group had greater behavioral deficits in self-care.
Wolf et al. (1999) [64]	DEU	Clinical	HRT+ (21) Placebo (17)	Cognitive tests: VFT, city maps task, Paired Associated Test, Stoop Test, mental rotation, mood assessment	17-β Estradiol	0.1	Percutaneously	Perimenopausal and Postmenopausal	HRT+ 69.5 ± 1.4 Placebo 67.8 ± 1.2	2 weeks	The treatment group analysis showed that women who received HRT and reached higher estradiol levels performed significantly better after treatment on delayed recall of paired associate tests. As these effects were seen after 2 weeks in women who were ~17 years postmenopausal, the findings of this study suggest that the brain remains sensitive to HRT where mediated changes occur rapidly.
LeBlanc et al. (2007) [22]	USA	Clinical	HRT+ (14) Placebo (18)	Cognitive tests: Oregon Sleep Health Diary, profile of mood states, paragraph recall and Verbal Paired Associates, facial emotion, abstract painting recall, letter category’s	17-β Estradiol	2	Oral	Postmenopausal	HRT+ 53.26 ± 0.64 Placebo 52.08 ± 0.64	8 weeks	Women receiving HRT did not have any improvements in cognitive performance or mood compared with those receiving placebo.
Maki et al. (2007) [65]	USA	Clinical	HRT+ (78) Placebo (80)	Cognitive tests: CVLT, (MFQ), BTA, Benton Visual Retention Test, Educational Testing Service Card rotation Test, Letter Fluency Test, WMS-R/LM-R, PANAS, The Greene Climacteric Scale, The Utian Quality of Life Scale, McCoy Female Sexuality Scale Questionnaire, PSQI.	CEE + MPA	0.625 + 2.5	Oral	Postmenopausal	45–55	4 months	There were no differences between the HRT group and placebo on any measures of cognition or quality of life. Modest negative effects on short-term and long-term verbal memory were observed but did not reach significance.
Alhola et al. (2010) [66]	FIN	Clinical	PreMP HRT+ (8) PreMP Placebo (8) Post PM HRT+ (7) Placebo (9)	Cognitive tests: Simple Reaction Time, Two Choice Reaction Time, 10 Choice Reaction Time, Subtraction task, Verification task, vigilance task, WAIS-R, PASAT, Bourdon–Wiersma Test, modified Rey Auditory Verbal Learning Test, Benton Visual Retention test.	Estradiol Valerate + Norethisterone	2 mg + 1 (0.7)	Oral	Premenopausal and Postmenopausal	PreMP 47.9 ± 1.7 PostMP 62.9 ± 2.9	6 months	In comparison to baseline, premenopausal women improved in tests of reaction time with the use of HRT, while the placebo group performed better in tests of attention. In postmenopausal women, HRT use was associated with improved performance in verbal episodic memory and minor declines in auditory attention.
Henderson et al. (2016) [67]	USA	Clinical	Early HRT+ (271) Late HRT+ (372)	Cognitive tests: SDMT, Complex Scanning and Visual Tracking, Trail Making Test (B), Shipley Institute of Living Scale, Abstraction Scale, Concept Formation, Letter Number Sequencing, Block Design, Animal Naming, WTAR, BNT, CVLT-s, EBMT	CEE ± MPA	1 ± 45	Oral Vaginal(Gel)	Postmenopausal	Early 55.4 ± 4.1 Late 65.4 ± 6.0	57 months	Results from the early and late groups did not differ significantly. Progesterone levels were significantly and positively associated with verbal memory and global cognition in early HRT initiation group. No evidence was found to support temporal proximity to menopause as a modifier of cognitive effect.
Perfanco et al. (2007) [68]	USA	Clinical	HRT+ (32) Placebo (25)	Cognitive tests: COWAT, Trail Making Test, WCST, BNT, the Digital Symbol Modalities Test, the Complex Figure Test, the Fold Object Memory Test, WMSIII, GDS, Beck Anxiety Inventory.	17-β Estradiol ± cyclic micronized progesterone	0.25 ± 100	NR	Postmenopausal	HRT+ 76 ± 6 Placebo 75 ± 4	3 years	No differences were found between groups on any neurocognitive or depression scores. Additionally, no differences were found when stratifying according to age.
Joffe et al. (2006) [69]	USA	Clinical	HRT+ (26) Placebo (26)	fMRI Cognitive tests: CVLT, WMS-R, Rey–Osterreith Complex Figure Test, PSQI, Beck Depression Inventory.	Micronized 17-β Estradiol ± micronized progesterone	0.05	TD	Perimenopausal and Postmenopausal	40–60 HRT+ 50.8 ± 3.4 Placebo 51.3 ± 4.2	12 weeks	HRT selectively reduced errors of preservation during verbal recall compared to placebo without influencing any other cognitive processes. Women with higher baseline scores of hot-flash severity demonstrated a greater cognitive benefit of HRT. Measures of fMRI BOLD activation during test of verbal and spatial working memory showed significant increases in frontal activity with HRT.
Kang and Grodstein (2012) [70]	USA	Clinical	Non HRT Users (5075) Past HRT users (5765) Current HRT unopposed Users (4188) Current HRT opposed users (1486)	Cognitive tests: TICS, EBMT-a, MMSE, Category Fluency, delayed recall of word list, digit span backwards	NR	NR	NR	Postmenopausal	70–81	26 years	In comparison to never users, past or current users of HRT showed worse rates of decline in the TICS. No protective association was found with early timing of initiation.
Shaywitz et al. (1999) [71]	USA	Clinical	46 (Crossover design)	fMRI Cognitive tests: verbal and non-verbal memory tasks	CEE	1.25	Oral	Postmenopausal	33–61 Mean 50.8 ± 4.7	21 days	HRT in a therapeutic dosage alters brain activation patterns in specific brain regions. However, HRT did not affect the performance score of verbal or non-verbal memory tasks.
Sherwin (1988) [72]	USA	Clinical	Climacteron (10) Estradiol Valerate (10) Testosterone enanthate (10) Placebo (10) Hysterectomy control (10)	Cognitive tests: Digit Span, WMS, Clerical Speed and Accuracy (Differential Aptitude Test), paragraph recall, Abstract Reasoning subset of DAT.	Climacteron (Testosterone, Estradiol dienanthate, Estradiol benzoate) Estradiol Valerate Testosterone enanthate	(150,7.5,1.0) 10 200	Intramuscular	Surgically menopausal	36.6–45.4 years	6 months	Test scores on short-term, long-term memory, or logical reasoning were not different during post-op treatment phase of HRT to their preoperative performance. Oophorectomized women who received placebo had lower scores of cognitive functioning postoperatively with lower concentrations of plasma estradiol and testosterone. Women who underwent hysterectomy but maintained their ovaries showed stable cognitive performance and in circulatory sex steroid concentrations. These results suggest that the drastic change in endocrine milieu following surgical menopause may have a direct, albeit modest, effect on aspects of cognitive function.
Phillips and Sherwin (1992) [73]	USA	Clinical	HRT+ (10) Placebo (9)	Cognitive tests: WMS, Menopausal Index, Multiple Affect Check List	Estradiol Valerate	10 mg/month	Intramuscular Injection	Surgically menopausal	Mean 48.2 ± 4.7	3 months	Scores for immediate and delayed recall paired associates stayed at same level in HRT-treated women, whereas they decreased significantly in pre- to post-op in the placebo group.
Wroolie et al. (2015) [74]	USA	Clinical	Continued HRT (30) Discontinued HRT (24)	Cognitive tests: WAIS, Auditory Consonant Trigrams Total, BSRT first trial learning; WMS-III, Color Trails 1; DKEFS, BSRT, Benton Visual Retention Test, Rey–Osterrieth Complex Figure Test, DKEFS, Memory Functioning Questionnaire	17-β Estradiol CEE	NR	NR	Postmenopausal	49–69 Discontinued 57.9 ± 5.01 Continued 58.2 ± 4.37	>3 years	Among women who were selected on a basis for a higher risk of AD and current HRT use, the continuation of HRT was associated with increased performance on cognitive domains, including verbal memory, combined attention, working memory and processing speed measures at the end of the follow-up period. All female patients diagnosed with AD declined in verbal memory; however, women who continued HRT declined less than women who discontinued HRT.
Silverman et al. (2011) [75]	USA	Clinical	17B Estradiol (35) CEE (18)	FDG Pet scan Cognitive tests: Auditory Consonant Trigrams, Benton Visual Retention Test, BNT, Color Trail Making Test, DKEFS, Rey–Osterrieth Complex Figure Test, WAISIII, WMS 3, Memory Function Questionnaire.	CEE 17-β Estradiol (Unopposed and Opposed)	NR	NR	Postmenopausal	50–65	2 years	Women taking 17-β estradiol performed better in verbal memory tasks than those taking CEE, where verbal memory performance positively correlated with metabolism in Wernicke’s and auditory association areas. Women taking opposed HRT had lower metabolism than women taking unopposed HRT in some brain regions.
Shumaker et al. (2003) [24]	USA	Clinical	HRT+ (2229) Placebo (2303)	MCI and AD diagnosis Cognitive tests: 3MSE, CERAD, BNT, word list memory task, contractional praxis, executive function.	CEE + MPA	0.625 + 2.5	Oral	Postmenopausal	65+	Mean 4.05 years	Opposed HRT increased the risk for probable dementia.
Grady et al. (2002) [76]	USA	Clinical	HRT+ (517) Placebo (546)	Cognitive tests: 3MSE, VFT, BNT, Word List Memory, Word List Recall, + Trials B)	CEE + MPA	0.625 + 2.5	Oral	Postmenopausal	Mean 71 ± 6	4 years	There were no differences in cognitive function performance between groups when adjusted for age in women with a history of coronary disease. However, women assigned to HRT scored worse on the VFT than women assigned to placebo.

## Data Availability

Not Applicable.
